# The genetic architecture of growth traits in *Salix matsudana* under salt stress

**DOI:** 10.1038/hortres.2017.24

**Published:** 2017-06-14

**Authors:** Jian Zhang, Huwei Yuan, Qingshan Yang, Min Li, Ying Wang, Yujuan Li, Xiangjian Ma, Feng Tan, Rongling Wu

**Affiliations:** 1 Center for Computational Biology, College of Biological Sciences and Technology, Beijing Forestry University, Beijing 100083, China; 2 Jiangsu Riverine Institute of Agricultural Sciences, Nantong, Jiangsu 226541, China; 3 State Key Laboratory of Subtropical Silviculture, School of Forestry & Bio-technology, Zhejiang A & F University, Hangzhou, Zhejiang 311300, China; 4 Center for Cultivation of Subtropical Forest Resources (CCSFR), School of Forestry & Bio-technology, Zhejiang A & F University, Hangzhou, Zhejiang 311300, China; 5 Shandong Academy of Forestry, Jinan, Shandong 250014, China; 6 Center for Statistical Genetics, Pennsylvania State University, Hershey, PA 17033, USA

## Abstract

Willow (*Salix*) is one of the most important ornamental tree species in landscape plants. One species, *Salix matsudana*, is widely used as a shade tree and border tree because of its soft branches and plump crown. Some varieties of *S. matsudana* were salt tolerant and could grow normally in coastal regions. However, the molecular mechanisms of salt tolerance for *S. matsudana* have been less clear. Here, we addressed this issue by performing a mapping experiment containing 195 intraspecific F_1_ progeny of *S. matsudana*, derived from salt-sensitive ‘yanjiang’ and salt-tolerant ‘9901’, grown by cuttings in a 100 mM NaCl solution. Growth performance of these progeny under salt stress was investigated, displaying marked genotypic variability with the coefficients of variance of 28.64–86.11% in shoot and root growth traits. We further mapped specific QTLs contributing to these differences to the *Salix* genome. Of the 204 QTLs identified, a few were detected to explain a remarkably larger portion of the phenotypic variation than many others. Many detected QTLs were found to reside in the region of candidate genes of known biological function. The discovery of growth QTLs expressed under salt stress provides important information for marker-assisted breeding of salt tolerant *Salix* varieties and founds the basis for the application of *S. matsudana* in coastal afforestation.

## Introduction

Willows (*Salix*), originated from China, have a wide distribution and strong adaptability.^1^ Willows are one of the major landscaping greening tree species and have played important roles in the ornamental of roads, courtyards, gardens and lakes. To date, some varieties of willows are widely planted in ecological environment construction of coastal areas and have showed good growth traits.

Because of their greening roles, willows have received considerable attention of genetic research. QTL mapping has been used to map specific loci that control biomass growth, developmental, physiological and biochemical traits. For example, Berlin *et al.* detected 60 QTLs associated with biomass and nitrogen economy traits in the hybrid population of *S. viminalis* and *S. schwerinii* using SNP markers, and each of these QTLs could explain 7.7–41.9% of the phenotypic variation.^2^ Ghelardini *et al.* further identified 80 QTLs responsible for growth arrest, leaf senescence and bud burst in the same population, and the most important QTL could explain 34% of the phenotypic variation for leaf senescence.^3^ Brereton *et al.* found QTLs related with enzymolysis saccharify on the 5th, 10th, and 11th linkage groups of ‘Björn’ (*S. viminalis*×*S. schwerinii*) and ‘78183’ (*S. viminalis*) hybrid population, and the highest contribution of QTL was 56.2%.^4^ In a mapping study of *S. purpurea* L. variety using RAPD markers, major QTLs for salicin were characterized, with the total contribution of 88.8%.^5^ As an important trait, pest resistance has been studied extensively in willow. Hoglund *et al.* have detected major QTLs for insect resistance in *S. viminalis* using SNP markers, with the contribution of 18%.^6^ One major QTL and 14 small QTLs have been found in the backcross population of *S. viminalis*×*S. schwerinii* and *S. viminalis* using SSR and SNP markers.^7^ All of the above studies have helped to reveal the genomic locations of genes controlling important traits of willow, and provide important references for marker-assisted breeding of willow.

*S. matsudana* is one of the most willow species that belongs mainly to allotetraploid.^8^ *S. matsudana*, originated from Northeast China, is named Chinese willow. Because of its strong adaptability and stress tolerance, *S. matsudana* had been introduced to Australia, Europe and North America^9^ used for greening and afforestation.^10^ *S. matsudana* plays important roles in saline land greening and soil heavy metal repair.^11^ Some varieties of *S. matsudana*, such as *S. matsudana* ‘Hailiu 1’ and *S. matsudana* ‘Yanjiang’, have become species with great potential in the greening of coastal city development of China.^12,13^ Because of the irreplaceable ecological status of willow in coastal areas, we have made tremendous efforts to study its genetic basis of complex traits. A SNP marker-based high density genetic map of tetraploid *Salix matsudana* has been constructed using its F_1_ hybrid population derived from ‘Yanjiang’ (female parent, a salt-sensitive variety in Jiangsu riverine regions of China) and ‘9901’ (male parent, a salt-tolerant variety selected in Shandong coastal regions of China).^8^ In this study, we report on the genetic architecture of growth traits for this mapping population grown under salt stress. A number of QTLs have been identified and mapped to the *Salix* genome, whose function was analyzed by comparing the corresponding candidate homologous genes in *Populus trichocarpa*. Results from this study will potentially provide guidance for marker-assisted breeding and cloning of genes controlling salt tolerant growth traits of *S. matsudana* and also have significance for environmental construction in coastal areas.

## Materials and methods

### Plant material

*S. matsudana* ‘Yanjiang’ (female parent, a salt-sensitive variety of *S. matsudana* in Jiangsu riverine regions of China) and *S. matsudana* ‘9901’ (a salt-tolerant variety of *S. matsudana* selected in Shandong coastal regions of China) were artificially crossed in the greenhouse in the winter of 2014. Hybrid seeds of F_1_ generation were collected and sowed early spring in 2015. A total of 195 hybrid seedlings of F_1_ generation were selected randomly and used as a population to construct a high-density genetic map.^8^ Cuttings, with diameters 2–3 cm and lengths 8–10 cm, were collected from the seedlings of the mapping population in August 2016 and cultured in the solution containing 100 mM NaCl. Each seedling has 30 cuttings, cultured in 3 transparent cups, with 10 cuttings in each cup. The cuttings were cultured in the illumination incubator with the photoperiod of 12 h day/12 h night, light intensity of 3300 Lux, temperature of 25 °C (the above culture method has been granted a patent, with the patent No. of ZL201210160422.6).

### Trait measurement

A total of 15 growth traits of the cuttings were measured, which can be divided into aboveground traits and underground traits. The 11 aboveground traits included start sprout days (SSD), branching angle of the first branch (AN), total sprout length (TSL), total leaf number (TLN), expand leaf number (ELN), nonexpand leaf number (NELN), expand leaf length (ELL), expand leaf width (ELW), leaf fresh weight (LFW), leaf dry weight (LDW) and content of chlorophyll (CH); the four underground traits include start root days (SRD), total root number (TRN), longest root length (LRL) and total root length (TRL).

SSD, TLN, ELN, NELN, SRD and TRN were observed and recorded directly, among which SSD and SRD were observed initially after 3 days of cultivation and observed once a day in the following days. AN was measured using protractor; TSL, ELL, ELW, LRL and TRL were measured using a tape; LFW was measured using electronic scales; the fresh leaves were dried in the drying oven at 105° for 3 h and then at 80° for 48 h, the dried leaves were collected and measured using electronic scales; CH was measured using SPAD-502 plus chlorophyll meter made in Japan. All the traits were measured within 20 days.

### Statistical analysis

SPSS 19.0 software was used to analyze the phenotypic variation of 15 traits and their phenotypic correlations. QTL mapping and contribution calculation were conducted using MapQTL6 software. The method of QTL mapping was Interval Mapping.

## Results

### Variation and correlation in growth traits under salt stress

**Figure 1:**
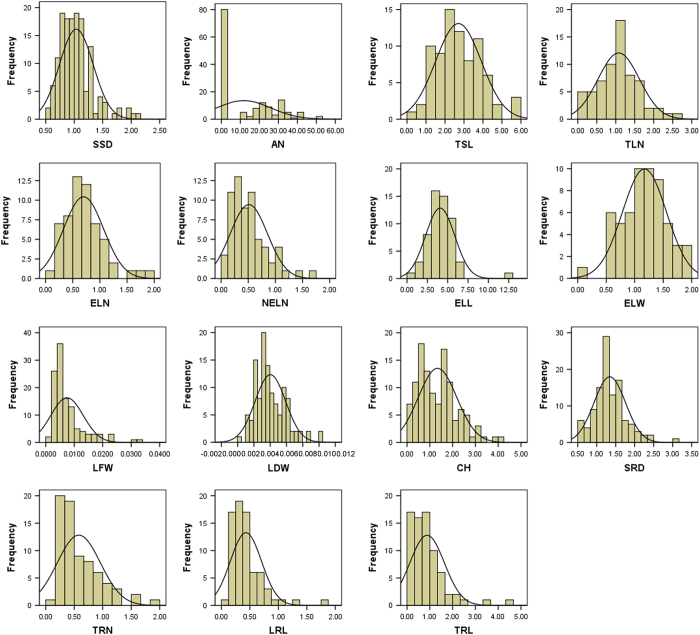
Histogram of frequency distribution for 15 growth traits of S. matsudana offsprings under salt stress. SSD and TSL represent start sprout days and total sprout length, respectively; AN represents branching angle of the first branch; TLN, ELN and NELN represent total leaf number; expand leaf number and non-expand leaf number, respectively; ELL, ELW, LFW, LDW and CH represent expand leaf length, expand leaf width, leaf fresh weight, leaf day weight and content of chlorophyll, respectively. SRD, TRN, LRL and TRL represent start root days, total root number, longest root length and total root length, respectively. These comments apply to other figures and tables as well.

All 15 growth traits expressed under salt stress showed approximately normal distributions (Figure 1). Skewness of these traits ranged from 0.0738 to 2.383 (Table 1), meaning that all the traits belong to a positively skewed normal distribution. Results from variation analysis showed that 15 growth traits had varying degrees of variation, with the coefficients of variation of 28.64–86.11%. Among the 15 growth traits measured, SSD and SRD had similar coefficients of variation of about 30%, demonstrating that the time differences of rooting and sprouting under salt stress were relatively small. The coefficient of variation for TRL was the highest (86.11%) among the 15 traits, meaning that TRL might be strongly influenced by salt stress.

Results from correlation analysis of the growth traits of *S. matsudana* offsprings under 100 mM NaCl treatment (Table 2) showed that SSD and SRD were positively correlated; LRL, TRN and TRL were positively correlated with each other; TSL, LFW, LDW and CH were positively correlated with each other; ELL, ELW, LFW, LDW and CH were positively correlated with each other.

### QTL mapping for growth traits under salt stress

On the basis of the phenotypic data of the 15 growth traits of *S. matsudana* offsprings, genes controlling these traits were mapped to the previously constructed intraspecific genetic map of tetraploid *S. matsudana*. A total of 204 QTLs were detected, with the LOD thresholds of 3.00–5.98, with the contributions ranging from 9.90 to 55.70% (Table 3). The QTLs were named under the following roles: start with letter ‘q’ which represents QTL; followed by the abbreviation of the corresponding trait; end with the number of the linkage group(s) on which the trait was mapped and the marker No. on the linkage group (s). QTLs of the 15 growth traits were summarized as follows:

- *SSD*: A total of four QTLs were detected, among which one was mapped on the 17th linkage group and the other three mapped on the 33rd linkage group. The maximum contribution was 11.00% for qSSD31.1.
- *AN*: A total of 19 QTLs were detected on the linkage groups of 10, 24, 33 and 38, among which nine QTLs were mapped on the 33rd linkage group. The maximum contribution was 26.00% for qAN10.2.
- *TSL*: A total of six QTLs were detected on the linkage groups of 2 and 21, with the contributions of 20.10–23.20%, among which the maximum contribution was qTSL21 mapped on the 21st linkage group.
- *TLN*: A total of six QTLs were detected, among which five were mapped on the 14th linkage group, another QTL (qTLN19) was mapped on the 19th linkage group, with the highest contribution of 23.50%.
- *ELN*: A total of 58 QTLs were detected on the linkage groups of 8, 14, and 18, among which 37 were distributed on the 8th linkage group. qELN8.37 had the highest contribution, which could explain 53.70% of the phenotypic variation.
- *NELN*: A total of five QTLs were detected on linkage group 2 and 13, among which 3 QTLs on the 13th linkage had contributions of >50.00%, with the highest value of 55.40% for qNELN13.1.
- *ELL*: One QTL was detected on linkage group 10, with the contribution of 22.90%.
- *ELW*: A total of 28 QTLs were detected on the linkage groups of 12, 15 and 28, among which qELW12.8 had the highest contribution of 34.90%.
- *LFW*: A total of three QTLs were detected on linkage group 32, with the highest contribution of 22.40%.
- *LDW*: A total of 19 QTLs were detected on linkage groups 11, 29 and 30. The highest contribution was 14.20% for qLDW11.2, qLDW11.3 and qLDW11.4.
- *CH*: A total of 14 QTLs were detected on linkage groups 6, 10 and 13. The highest contribution was 15.40% for qCH10.3.
- *SRD*: A total of 15 QTLs were detected on linkage groups 17, 21, 32 and 33, with the contribution ranges of 12.50–18.60%.
- *TRN*: One QTL was detected on linkage group 16, with the contribution of 55.70%.
- *LRL*: A total of 13 QTLs were detected on linkage groups 28, 36 and 38. The highest contribution was 25.90% for qLRL38.
- *TRL*: A total of 12 QTLs were detected on linkage groups 3, 9, 12 and 16. The lowest and highest contributions were 18.40 for qTRL12.4 and 55.70% for qTRL16.

Detailed QTL mapping results of the 15 growth traits are listed in Table 3.

**Figure 2:**
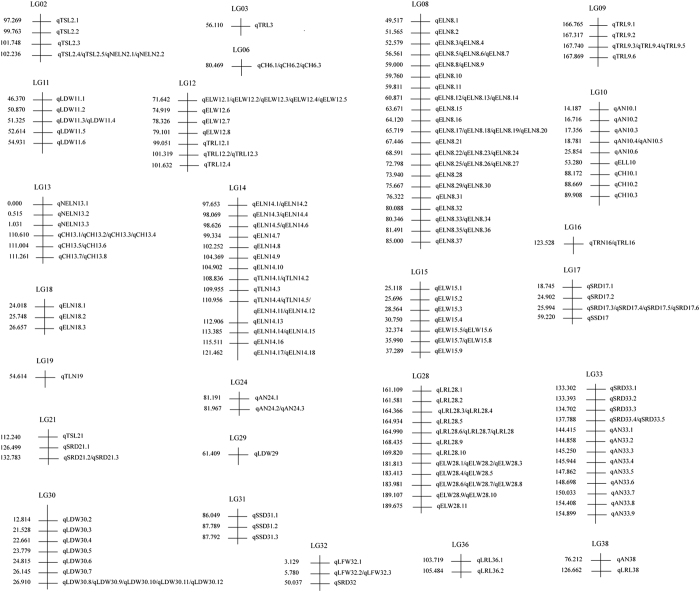
Location of QTLs detected on the linkage groups of the F_1_ population (*S. matsudana* ‘yanjiang’×*S. matsudana* ‘9901’) for 15 growth traits of *S. matsudana* offsprings under salt stress.

*S. matsudana* belongs to a willow species of allotetraploid (2*n*=4x=76), the genetic map of which consisted of 38 linkage groups.^8^ Locations of QTLs for 15 growth traits of *S. matsudana* offsprings under salt stress are shown in Figure 2. As can be seen, the 204 QTLs detected were located on 25 linkage groups, 2, 3, 6, 8–19, 21, 24, 28–33, 36 and 38. QTLs for TSL and NELN were located on linkage group 2; QTLs for AN, ELL and CH were located on linkage group 10; QTLs related with ELW and TRL were located on linkage group 12; QTLs related with NELN and CH were located on linkage group 13; Genes controlling TLN and ELN were on linkage group 14; The regions controlling SSD and SRD were located on linkage group 17; Regions controlling TSL and SRD were located on linkage group 21; Linkage group 28 contained regions controlling LRL and ELW; Genes controlling LFW and SRD were located on linkage group 32; There were regions controlling SRD and AN on linkage group 33; Linkage group 38 contained regions controlling AN and LRL. Meanwhile, locus 102.236 on linkage group 2 contained 4 QTLs controlling TSL and NELN; locus 110.956 on linkage group 14 contained 4 QTLs controlling TLN and ELN, implying that these loci are pleiotropic QTLs, each controlling multiple traits.

In addition, the genetic map was constructed using SLAF-seq technique, whose length of sequence tags was about 200 bp.^14^ As most genes had the length of about or >1 kb, adjacent QTLs detected in this study might belong to one or more genes. Consequently, we tested segregation profiles of QTLs controlling the same trait on the same linkage group in the F_1_ offspring of *S. matsudana*. Results showed that three QTLs on linkage group 6 (qCH6.1, qCH6.2 and qCH6.3) are segregating among the 195 offspring in the same fashion, suggesting that these three QTLs may represent a single locus. A similar result was obtained for two QTLs on linkage group 15 (qELW15.5 and qELW15.6), two QTLs on linkage group 28 (qLRL28.1 and qLRL 28.2), and two QTLs on linkage group 31 (qSSD31.2 and qSSD31.3).

### Functional analysis of candidate homologous genes of *S. matsudana* QTLs in *Populus trichocarpa*

**Figure 3:**
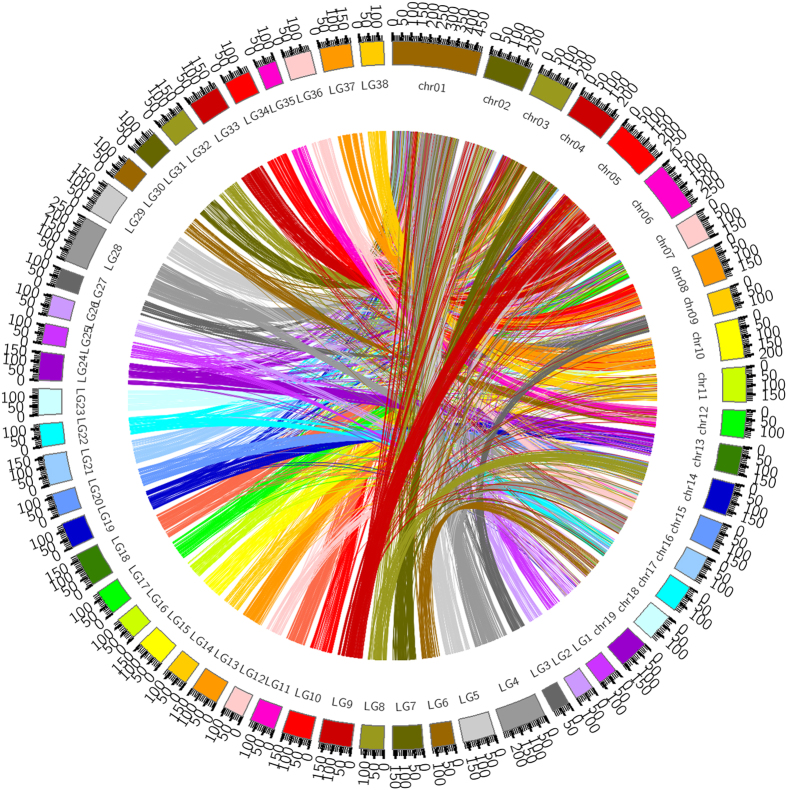
Colinear relationships between *S. matsudana* linkage group and *P. trichocarpa* chromosomes.

Willow and poplar belong to Salicaceae. They had evolved from the same ancestor and had many homologous fragments in their genomes. As the genomes of willow have not been released to public, while the genome of *P. trichocarpa* has been well studied,^15^ we aligned the high density genetic map of *S. matsudana* with the genome of *P. trichocarpa*. Results showed that the 38 linkage groups of *S. matsudana* had different correspondence with the chromosomes of *P. trichocarpa*. The homologous fragments for all 38 linkage groups of *S. matsudana* could be found in the genome of *P. trichocarpa*, demonstrating high homology between the genomes of *S. matsudana* and *P. trichocarpa* (Figure 3).

**Figure 4:**
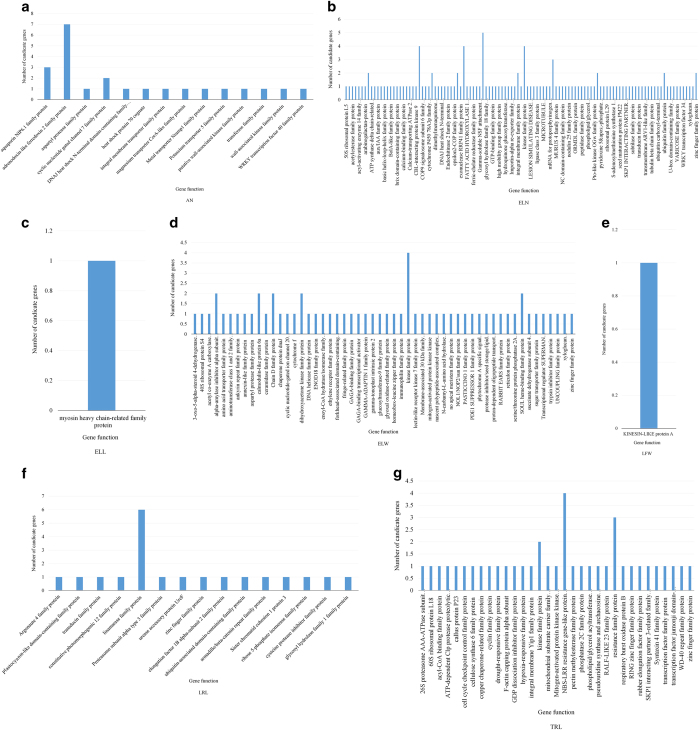
Functional analysis of candidate homologous genes of *S. matsudana* QTLs in *P. trichocarpa* chromosomes. Note: Explanation of the trait abbreviations are shown in the comments of Figure 1.

To further predict the function of genes associated with QTLs of the 15 growth traits of *S. matsudana* offsprings under salt stress, we searched for all genes in the genome regions of *P. trichocarpa* that are homologous with 204 QTLs in *S. matsudana*, and analyzed possible functions of these genes through functional annotation based on NR database. Results showed that genes with multiple functions have been found in the genome regions of *P. trichocarpa* that are homologous with QTLs related with seven out of the 15 growth traits, including AN, ELN, ELL, ELW, LFW, LRL and TRL, in *S. matsudana* (Figure 4). These genes might have important roles in the processes of ion transport, energy conversion, signal transduction, protein metabolism, water transportation, stress resistance and biomembrane repair.

## Discussion

### Growth performance under salt stress

As one of the most important willow species, *S. matsudana* is widely distributed and grown in China. Divergent habitat differences in the distribution of *S. matsudana* have contributed to extensive adaptabilities in the long term evolution process of this species. To explore the possibility of *S. matsudana* to be grown in coastal areas, some researchers had investigated its salt tolerance and selected some salt-tolerant varieties of this species.^12,13^ However, the molecular mechanisms underlying the salt tolerance of *S. matsudana* have been less studied, which has restricted efficient selection of more salt tolerant *S. matsudana* varieties. QTL mapping of important economic traits for *S. matsudana* is a powerful tool to reveal the molecular mechanisms of salt tolerance for this species, which could provide a basis for map-based cloning and marker-assisted breeding.

QTL mapping is a technique that can find the genome locations of genes controlling quantitative traits by combining phenotypic variation of traits with marker-based genetic map.^16,17^ Variation in traits is the basis for successful QTL mapping of the target traits. To efficiently conduct QTL mapping, we measured 15 growth traits of cuttings from the 195 F_1_ offspring of salt-sensitive *S. matsudana* ‘Yanjiang’×salt-tolerant *S. matsudana* ‘9901’ under 100 mM-salt stress, and variation in these traits was analyzed. Results showed that these traits displayed approximately normal distributions (Figure 1), and coefficients of variance for the 15 growth traits ranged from 28.64 to 86.11% (Table 1). According to the previous definition, the variation of a trait is significant if coefficient of variance for the trait is >10%.^18^ In this study, all the 15 growth traits had the coefficients of variance of significantly higher than 10%, demonstrating that there were significant variations among different genotypes in these traits. Significant variation in the growth traits of *S. matsudana* offspring under salt stress has provided the basis for QTL mapping of these traits.

Correlations between the 15 growth traits of *S. matsudana* offsprings under salt stress showed that SSD and SRD were positively correlated (Table 2), demonstrating that rooting and sprouting were two mutually promoted growth processes. LRL, TRN, TRL were positively related with each other (Table 2), implying that the increase of root number contribute to the rise in root length. TSL, LFW, LDW and CH were significantly related with each other (Table 2), implying that the growth of sprout and leaves is complementary to each other. There were significant correlations between ELL, ELW, LFW, LDW and CH (Table 2), showing that the growth of leaf traits promoted each other. These results were in accordance with the previous results about correlations between growth traits in *Prunus mume* and *Portulaca oleracea*.^19,20^

### QTL mapping of growth traits under salt stress

Results from QTL mapping for the F_1_ hybrid population of *S. matsudana* under salt stress showed that a total of 204 QTLs were detected for 15 growth traits with the LODs of >3.00 and the contributions of >9.90% (Table 3). These QTLs were located on 25 linkage groups of the genetic map, each of which could explain 9.90–55.70% of the corresponding trait variation (Table 3). Among the 15 growth traits, 3 QTLs on linkage group 18 were related with ELN, with the contributions of 51.20%, 50.40% and 48.70%, respectively (Figure2 and Table 3); 3 QTLs on linkage group 13 were related with NELN, with the contributions of 55.40%, 55.20% and 54.90%, respectively (Figure 2 and Table 3); 1 QTL related with TRN were found on linkage groups 3 and 16, with the contributions of 55.10% and 55.70%, respectively (Figure 2 and Table 3). These results showed that there were genes on these linkage groups that control some growth traits of *S. matsudana* under salt stress.

It has been found that QTLs associated with different traits were located within the same region of the same linkage group. For example, QTLs related with LRL and ELW were located in the same regions between 161.109 and 189.675 on linkage group 28 (Figure 2), and QTLs related with SRD and AN were located in the same regions between 133.302 and 154.899 on linkage group 33 (Figure 2). It is common that QTLs controlling different traits were located in the same regions. For instance, QTLs controlling the growth and wood quality traits of *Eucalyptus* ^21^ were located in the same regions. Such so-called pleiotropic QTLs were also observed for different growth traits in willow,^22,23^ different leaf morphology traits in oak ^24^ and different chemical component related traits in peach and oil palm.^25,26^ Pleiotropic effects were believed to be an important component for the genetic architecture of quantitative traits.^27,28,29^

### Functional analysis of growth QTLs under salt stress

Willow and poplar are all species in Salicaceae. They had evolved from the same ancestor and had many homologous fragments in their genomes.^30,31^ As the genomes of willow have not been released to public, functions of candidate genes associated with QTLs controlling the growth traits of *S. matsudana* under salt stress were predicted according to the genome of *P. trichocarpa*. Results from colinear analysis between the linkage groups of *S. matsudana* and the chromosomes of *P. trichocarpa* showed that the 38 linkage groups of *S. matsudana* had different correspondence with the chromosomes of *P. trichocarpa*. The homologous fragments for all 38 linkage groups of *S. matsudana* could be found in the genome of *P. trichocarpa* (Figure 3), demonstrating high homology between the genomes of *S. matsudana* and *P. trichocarpa* and functions of candidate genes associated with QTLs in *S. matsudana* might be predicted based on the genome information of *P. trichocarpa*.

In this study, genes in the homologous regions of QTLs controlling the growth traits of *S. matsudana* were searched in *P. trichocarpa*. Finally, genes in the homologous regions of QTLs controlling seven growth traits of *S. matsudana* were found in *P. trichocarpa*, obtaining functional annotation successfully. For example, QTLs controlling LFW may reside in the homologous regions of genes encoding kinesis-like protein A (Figure 4e). Similarly, we found that QTLs controlling ELW may be related to genes encoding amino acid transporter family protein and DNA helicase family protein (Figure 4d), QTLs controlling ELN related to genes encoding ATPase family protein and aux/IAA family protein (Figure 4b), and QTLs controlling TRL related to genes encoding callus protein P23 and cell cycle checkpoint control family protein (Figure 4g). These genes might have important roles in the processes of ion transport, energy conversion, signal transduction, protein metabolism, water transportation, stress resistance and biomembrane repair. We predicted therefore that the metabolism was vigorous during the growth of *S. matsudana* after salt treatment, which might be controlled by multiple genes with varying functions.

However, since willow and poplar have undergone divergent evolution processes, there must be great differences between their genomes. The exact function of genes associated with QTLs controlling the growth traits of *S. matsudana* under salt stress could only be verified based on the genome information of *S. matsudana*.

## Conclusions

Using cuttings collected from 195 F_1_ hybrid offsprings of salt-sensitive *S. matsudana* ‘Yanjiang’×salt-tolerant *S. matsudana* ‘9901’ as a mapping population, variation and correlations among 15 growth traits of the cuttings cultured in 100 mM NaCl solution were analyzed. QTLs controlling these growth traits were mapped on the linkage groups and gene functions located in the QTLs were predicted. We found significant variation among all the traits measured. Some growth traits were positively correlated with each other. A total of 204 QTLs were detected for the 15 growth traits. Genes located in these QTLs might have different functions and participate in different processes of growth and development.

## Figures and Tables

**Table 1:** Statistical analysis of 15 growth traits for offsprings of *S. matsudana* under salt stress

*Trait No.*	*Trait*	*Min*	*Max*	*Mean*	*SD*	*SK*	*CV (%)*
1	SSD	0.51	2.13	1.04	0.297	1.291	28.64
2	AN	10	50	26	9.052	0.422	34.82
3	TSL (mm)	0.45	5.98	2.71	1.204	0.436	44.49
4	TLN	0.18	2.54	1.09	0.533	0.435	48.66
5	ELN	0.17	1.95	0.71	0.361	1.177	50.74
6	NELN	0.17	1.72	0.54	0.331	1.416	61.87
7	ELL (mm)	1.21	12.77	4.14	1.732	2.082	41.82
8	ELW (mm)	0.54	1.87	1.19	0.357	0.0738	29.93
9	LFW (g)	0.0016	0.0321	0.0075	0.00567	2.089	75.96
10	LDW (g)	0.0002	0.0102	0.0040	0.00187	0.860	46.61
11	CH	0.04	4.03	1.34	0.848	0.798	63.37
12	SRD	0.52	3.00	1.34	0.415	0.790	30.89
13	TRN	0.14	1.95	0.58	0.379	1.377	65.26
14	LRL (mm)	0.12	1.78	0.43	0.274	2.383	63.72
15	TRL (mm)	0.12	4.55	0.88	0.759	2.313	86.11

SD, SK and CV represent standard deviation, skewedness and coefficient of variation, respectively. Explanations of the trait abbreviations are shown in the comments of Figure 1.

**Table 2:** Correlations between 15 growth traits of *S. matsudana* offsprings under salt stress

	*SSD*	*AN*	*TSL*	*TLN*	*ELN*	*NELN*	*ELL*	*ELW*	*LFW*	*LDW*	*CH*	*SRD*	*TRN*	*LRL*	*TRL*
SSD	1	−0.116	−0.114	0.083	0.166	−0.005	−0.181	−0.123	−0.124	−0.225*	−0.009	0.489**	0.050	0.039	−0.017
AN	−0.083	1	0.356**	0.376**	0.210	0.147	−0.204	−0.047	0.273**	0.313**	0.178	−0.131	0.204	0.217	0.256*
TSL	−0.012	0.300**	1	0.235	0.101	0.066	0.227	0.098	0.307**	0.512**	0.403**	0.107	−0.075	−0.027	−0.108
TLN	0.127	0.275*	0.244	1	0.693**	0.661**	0.185	0.226	0.187	0.107	0.200	0.037	−0.109	0.103	−0.039
ELN	0.257	0.095	0.185	0.654**	1	0.003	0.017	0.090	0.303*	−0.115	0.256	0.219	−0.194	−0.228	−0.229
NELN	0.020	0.126	0.046	0.632**	−0.025	1	0.222	0.222	−0.056	0.107	0.007	−0.101	−0.127	0.319	0.033
ELL	−0.152	−0.322*	0.312*	0.215	0.063	0.254	1	0.623**	0.245	0.301*	0.300*	−0.091	−0.144	−0.216	−0.199
ELW	−0.074	−0.121	0.098	0.231	0.086	0.261*	0.763**	1	0.350*	0.394**	0.382**	0.036	−0.027	−0.247	−0.166
LFW	−0.126	0.400**	0.360**	0.170	0.256	−0.061	0.454**	0.367**	1	0.652**	0.204*	0.001	0.015	−0.071	−0.020
LDW	−0.173	0.358**	0.502**	0.036	−0.136	−0.017	0.447**	0.405**	0.780**	1	0.256**	−0.146	0.275*	0.364**	0.357**
CH	−0.028	0.217*	0.463**	0.233	0.227	0.111	0.301*	0.377**	0.321**	0.351**	1	0.035	0.043	−0.050	−0.028
SRD	0.379**	−0.064	0.163	−0.011	0.179	−0.092	−0.090	−0.023	−0.106	−0.149	−0.007	1	−0.134	−0.087	−0.185
TRN	0.059	0.155	0.066	−0.019	−0.191	−0.009	−0.116	0.016	0.151	0.268*	0.071	−0.135	1	0.514**	0.868**
LRL	0.143	0.094	0.069	0.048	−0.257	0.102	−0.337*	−0.234	−0.101	0.168	−0.032	−0.068	0.530**	1	0.820**
TRL	0.022	0.185	0.030	0.010	−0.297	0.083	−0.233	−0.109	0.069	0.294*	0.048	−0.203	0.869**	0.829**	1

Data above the diagonal represent Pearson correlation coefficients; data below the diagonal represent Spearman correlation coefficients. * and ** represent significant correlation at *P*<0.05 and *P*<0.01 probability levels, respectively. Explanations of the trait abbreviations are shown in the comments of Figure 1.

**Table 3:** QTL characterization of 15 growth traits for offsprings of *S. matsudana* under salt stress

*Trait*	*QTL No.*	*Linkage group*	*LOD threshold*	*Peak position*	*Marker*	*Contribution (%)*
SSD	qSSD17	17	3.05	59.220	Marker4417	9.90
	qSSD31.1	31	3.47	86.049	Marker68958	11.00
	qSSD31.2	31	3.20	87.789	Marker56033	10.70
	qSSD31.3	31	3.20	87.792	Marker12854	10.70
AN	qAN10.1	10	3.60	14.187	Marker21108	24.80
	qAN10.2	10	4.04	16.716	Marker233895	26.00
	qAN10.3	10	4.06	17.356	Marker11454	25.70
	qAN10.4	10	3.15	18.781	Marker102865	20.60
	qAN10.5	10	3.15	18.781	Marker21903	20.60
	qAN10.6	10	3.06	25.854	Marker91103	24.10
	qAN24.1	24	3.10	81.191	Marker111179	20.30
	qAN24.2	24	3.10	81.967	Marker135892	20.30
	qAN24.3	24	3.10	81.967	Marker82497	20.30
	qAN33.1	33	3.11	144.415	Marker23501	21.20
	qAN33.2	33	3.21	144.858	Marker50270	20.90
	qAN33.3	33	3.21	145.250	Marker127246	20.90
	qAN33.4	33	3.55	145.944	Marker33462	24.10
	qAN33.5	33	3.88	147.862	Marker85800	25.10
	qAN33.6	33	3.84	148.698	Marker96137	24.90
	qAN33.7	33	3.72	150.033	Marker19266	23.80
	qAN33.8	33	3.42	154.408	Marker31234	23.30
	qAN33.9	33	3.38	154.899	Marker26610	23.10
	qAN38	38	3.38	76.212	Marker50607	23.20
TSL	qTSL2.1	2	3.20	97.269	Marker8950	20.10
	qTSL2.2	2	3.33	99.763	Marker1698	20.50
	qTSL2.3	2	3.94	101.748	Marker2480	21.30
	qTSL2.4	2	4.13	102.236	Marker9052	21.90
	qTSL2.5	2	4.12	102.236	Marker36987	21.90
	qTSL21	21	3.36	112.240	Marker50353	23.20
TLN	qTLN14.1	14	3.09	108.836	Marker4468	20.60
	qTLN14.2	14	3.09	108.836	Marker182582	20.60
	qTLN14.3	14	3.11	109.955	Marker18056	20.70
	qTLN14.4	14	3.05	110.956	Marker198292	20.40
	qTLN14.5	14	3.05	110.956	Marker189568	20.40
	qTLN19	19	3.01	54.614	Marker12384	23.50
ELN	qELN8.1	8	3.01	49.517	Marker34843	31.40
	qELN8.2	8	3.24	51.565	Marker31326	32.80
	qELN8.3	8	3.23	52.579	Marker6145	32.10
	qELN8.4	8	3.22	52.579	Marker20403	32.10
	qELN8.5	8	3.04	56.561	Marker97091	22.90
	qELN8.6	8	3.04	56.561	Marker11847	22.90
	qELN8.7	8	3.04	56.561	Marker38261	22.90
	qELN8.8	8	4.55	59.000	Marker4180	32.90
	qELN8.9	8	4.55	59.000	Marker73106	32.90
	qELN8.10	8	4.66	59.760	Marker62726	32.80
	qELN8.11	8	4.66	59.811	Marker58460	32.80
	qELN8.12	8	4.55	60.871	Marker19509	32.50
	qELN8.13	8	4.55	60.871	Marker29948	32.50
	qELN8.14	8	4.55	60.871	Marker38483	32.50
	qELN8.15	8	4.39	63.671	Marker41206	31.20
	qELN8.16	8	4.42	64.120	Marker23303	31.40
	qELN8.17	8	3.74	65.719	Marker7707	27.40
	qELN8.18	8	3.72	65.719	Marker34875	27.20
	qELN8.19	8	3.72	65.719	Marker4196	27.20
	qELN8.20	8	3.72	65.719	Marker11304	27.20
	qELN8.21	8	3.40	67.446	Marker15084	26.30
	qELN8.22	8	3.20	68.591	Marker100182	25.60
	qELN8.23	8	3.20	68.591	Marker58121	25.60
	qELN8.24	8	3.19	68.591	Marker5391	25.60
	qELN8.25	8	3.22	72.798	Marker9488	24.60
	qELN8.26	8	3.22	72.798	Marker19137	24.60
	qELN8.27	8	3.22	72.798	Marker27106	24.60
	qELN8.28	8	3.36	73.940	Marker22173	25.00
	qELN8.29	8	3.37	75.667	Marker51402	25.10
	qELN8.30	8	3.37	75.667	Marker15477	25.10
	qELN8.31	8	3.42	76.322	Marker48946	25.40
	qELN8.32	8	3.72	80.088	Marker21104	27.20
	qELN8.33	8	3.75	80.346	Marker13673	27.70
	qELN8.34	8	3.75	80.346	Marker52362	27.80
	qELN8.35	8	3.82	81.491	Marker38676	30.30
	qELN8.36	8	3.82	81.491	Marker8778	30.30
	qELN8.37	8	4.46	85.000	Marker41145	53.70
	qELN14.1	14	3.59	97.653	Marker9790	27.00
	qELN14.2	14	3.60	97.653	Marker28984	27.00
	qELN14.3	14	3.61	98.069	Marker51357	27.00
	qELN14.4	14	3.61	98.069	Marker198204	27.00
	qELN14.5	14	3.63	98.626	Marker63666	27.00
	qELN14.6	14	3.63	98.626	Marker53190	27.00
	qELN14.7	14	3.66	99.334	Marker36205	27.20
	qELN14.8	14	3.53	102.252	Marker37914	27.00
	qELN14.9	14	3.33	104.369	Marker150911	26.00
	qELN14.10	14	3.28	104.902	Marker57901	25.70
	qELN14.11	14	3.05	110.956	Marker198292	23.30
	qELN14.12	14	3.06	110.956	Marker189568	23.40
	qELN14.13	14	3.39	112.906	Marker172810	25.30
	qELN14.14	14	3.46	113.385	Marker18119	25.50
	qELN14.15	14	3.46	113.385	Marker121309	25.50
	qELN14.16	14	3.01	115.511	Marker21515	22.90
	qELN14.17	14	3.05	121.462	Marker24440	23.20
	qELN14.18	14	3.05	121.462	Marker43327	23.20
	qELN18.1	18	3.81	24.018	Marker8078	51.20
	qELN18.2	18	3.76	25.748	Marker46742	50.40
	qELN18.3	18	3.31	26.657	Marker103743	48.70
NELN	qNELN2.1	2	3.00	102.236	Marker9052	20.90
	qNELN2.2	2	3.00	102.236	Marker36987	20.90
	qNELN13.1	13	3.19	0.000	Marker39456	55.40
	qNELN13.2	13	3.13	0.515	Marker5800	55.20
	qNELN13.3	13	3.08	1.031	Marker31858	54.90
ELL	qELL10	10	3.05	53.280	Marker84010	22.90
ELW	qELW12.1	12	3.61	71.642	Marker45696	26.50
	qELW12.2	12	3.61	71.642	Marker75852	26.50
	qELW12.3	12	3.61	71.642	Marker8765	26.50
	qELW12.4	12	3.61	71.642	Marker7025	26.50
	qELW12.5	12	3.62	71.642	Marker6425	26.60
	qELW12.6	12	4.41	74.919	Marker8589	32.60
	qELW12.7	12	4.69	78.326	Marker58205	34.60
	qELW12.8	12	4.71	79.101	Marker16205	34.90
	qELW15.1	15	3.36	25.118	Marker75603	26.60
	qELW15.2	15	3.41	25.696	Marker28450	26.60
	qELW15.3	15	3.22	28.564	Marker14011	24.00
	qELW15.4	15	3.85	30.750	Marker27094	29.10
	qELW15.5	15	3.13	32.374	Marker32487	25.30
	qELW15.6	15	3.12	32.374	Marker53886	25.30
	qELW15.7	15	3.02	35.990	Marker255588	23.40
	qELW15.8	15	3.02	35.990	Marker159162	23.40
	qELW15.9	15	3.18	37.289	Marker16036	24.10
	qELW28.1	28	3.10	181.813	Marker5090	23.80
	qELW28.2	28	3.10	181.813	Marker10335	23.80
	qELW28.3	28	3.10	181.813	Marker188421	23.80
	qELW28.4	28	3.30	183.413	Marker86604	26.10
	qELW28.5	28	3.30	183.413	Marker711	26.10
	qELW28.6	28	3.31	183.981	Marker23052	26.80
	qELW28.7	28	3.31	183.981	Marker25984	26.90
	qELW28.8	28	3.31	183.981	Marker249891	26.90
	qELW28.9	28	3.30	189.107	Marker25181	29.80
	qELW28.10	28	3.30	189.107	Marker80553	29.80
	qELW28.11	28	3.29	189.675	Marker13987	29.30
LFW	qLFW32.1	32	5.86	3.129	Marker21754	22.30
	qLFW32.2	32	5.98	5.780	Marker30807	22.40
	qLFW32.3	32	5.98	5.780	Marker242064	22.40
LDW	qLDW11.1	11	3.15	46.370	Marker179545	13.60
	qLDW11.2	11	3.66	50.870	Marker11487	14.20
	qLDW11.3	11	3.68	51.325	Marker196916	14.20
	qLDW11.4	11	3.69	51.325	Marker31756	14.20
	qLDW11.5	11	3.50	52.614	Marker109416	13.70
	qLDW11.6	11	3.17	54.931	Marker216401	13.00
	qLDW29	29	3.02	61.409	Marker8982	11.80
	qLDW30.1	30	3.04	9.287	Marker39654	12.70
	qLDW30.2	30	3.01	12.814	Marker46151	12.20
	qLDW30.3	30	3.33	21.528	Marker21155	13.50
	qLDW30.4	30	3.29	22.661	Marker146281	13.20
	qLDW30.5	30	3.24	23.779	Marker17960	12.90
	qLDW30.6	30	3.07	24.815	Marker34424	12.10
	qLDW30.7	30	3.19	26.145	Marker15294	12.50
	qLDW30.8	30	3.22	26.910	Marker59686	12.50
	qLDW30.9	30	3.22	26.910	Marker31564	12.50
	qLDW30.10	30	3.22	26.910	Marker31978	12.50
	qLDW30.11	30	3.22	26.910	Marker73135	12.50
	qLDW30.12	30	3.22	26.910	Marker44938	12.50
CH	qCH6.1	6	3.02	80.469	Marker37145	11.90
	qCH6.2	6	3.02	80.469	Marker177833	11.90
	qCH6.3	6	3.02	80.469	Marker60039	11.90
	qCH10.1	10	3.11	88.172	Marker12641	13.50
	qCH10.2	10	3.11	88.669	Marker229975	13.60
	qCH10.3	10	3.43	89.908	Marker156703	15.40
	qCH13.1	13	3.15	110.610	Marker9383	12.30
	qCH13.2	13	3.15	110.610	Marker27262	12.30
	qCH13.3	13	3.15	110.610	Marker2160	12.30
	qCH13.4	13	3.16	110.610	Marker29644	12.30
	qCH13.5	13	3.53	111.004	Marker7790	13.60
	qCH13.6	13	3.53	111.004	Marker41703	13.60
	qCH13.7	13	3.50	111.261	Marker1109	13.50
	qCH13.8	13	3.49	111.261	Marker756	13.50
SRD	qSRD17.1	17	3.20	18.745	Marker24340	15.10
	qSRD17.2	17	4.07	24.902	Marker247669	16.60
	qSRD17.3	17	3.93	25.994	Marker246323	15.80
	qSRD17.4	17	3.93	25.994	Marker45854	15.80
	qSRD17.5	17	3.93	25.994	Marker41634	15.80
	qSRD17.6	17	3.92	25.994	Marker147678	15.80
	qSRD21.1	21	3.37	126.499	Marker103086	18.60
	qSRD21.2	21	3.20	132.783	Marker109132	17.10
	qSRD21.3	21	3.20	132.783	Marker24927	17.10
	qSRD32	32	3.02	50.037	Marker53056	12.50
	qSRD33.1	33	3.41	133.302	Marker28619	13.80
	qSRD33.2	33	3.43	133.393	Marker158480	13.80
	qSRD33.3	33	3.40	134.702	Marker53313	13.70
	qSRD33.4	33	3.29	137.788	Marker6919	13.40
	qSRD33.5	33	3.27	137.788	Marker7854	13.30
TRN	qTRN16	16	4.90	123.528	Marker13996	55.70
LRL	qLRL28.1	28	3.43	161.109	Marker11431	21.60
	qLRL28.2	28	3.43	161.581	Marker22520	21.40
	qLRL28.3	28	3.44	164.366	Marker25131	20.50
	qLRL28.4	28	3.44	164.366	Marker10717	20.50
	qLRL28.5	28	3.44	164.934	Marker162057	20.50
	qLRL28.6	28	3.44	164.990	Marker11722	20.50
	qLRL28.7	28	3.44	164.990	Marker25926	20.50
	qLRL28.8	28	3.44	164.990	Marker166380	20.50
	qLRL28.9	28	3.46	168.435	Marker20175	21.90
	qLRL28.10	28	3.40	169.820	Marker36640	22.20
	qLRL36.1	36	3.05	103.719	Marker130938	18.80
	qLRL36.2	36	3.06	105.484	Marker59009	18.90
	qLRL38	38	3.33	126.662	Marker49640	25.90
TRL	qTRL3	3	3.75	56.110	Marker52503	55.10
	qTRL9.1	9	3.12	166.765	Marker30097	19.00
	qTRL9.2	9	3.19	167.317	Marker25601	19.30
	qTRL9.3	9	3.23	167.740	Marker95203	19.40
	qTRL9.4	9	3.23	167.740	Marker25663	19.40
	qTRL9.5	9	3.23	167.740	Marker32946	19.40
	qTRL9.6	9	3.22	167.869	Marker193289	19.40
	qTRL12.1	12	3.57	99.051	Marker6923	21.30
	qTRL12.2	12	3.16	101.319	Marker56910	19.50
	qTRL12.3	12	3.16	101.319	Marker191788	19.50
	qTRL12.4	12	3.05	101.632	Marker9305	18.40
	qTRL16	16	3.53	123.528	Marker13996	55.70

Explanation of the trait abbreviations are shown in the comments of Figure 1.
